# Retraction: Functional Role of mTORC2 versus Integrin-Linked Kinase in Mediating Ser473-Akt Phosphorylation in PTEN-Negative Prostate and Breast Cancer Cell Lines

**DOI:** 10.1371/journal.pone.0202299

**Published:** 2018-08-09

**Authors:** The PLOS ONE Editors

The Ohio State University Office of Research Compliance notified *PLOS ONE* that the institution investigated the work reported in this article [1] and found evidence of data falsification in Figures 1B, 3A, and 3B.

In Figure 1B, the p70S6K and β-actin Western blot data were replaced with unidentified blots, and the Akt blot for LNCaP cells was incorrectly labeled when compared to the original research record. No supporting data for the published blots for p70S6K or β-actin panels were identified in the investigation, while the published blot for the Akt panel was shown to be generated under different experimental conditions than as reported. These changes resulted in a figure that shows equal expression of p70S6K where the original data for the described experiment showed unequal expression.

In Figure 3A, Western blot data in the MK2 blot (lanes 1, 3) and the PAK1 blot (lanes 1, 3, 8) were replaced and relabeled when compared to the original research record. This changed the reported results by showing all the untreated samples in the MK2 and PAK1 blots had the same expression level, while the original research record showed unequal expression.

In Figure 3B, Western blot data for the p308-T-Akt, ILK, Akt and β-actin blots were incorrectly labeled as being treated with or without Ku-0063794 and/or ILK siRNA when compared to the original research record. This changed the reported results by showing that ILK knockdown had no effect on the expression of pSer473AKT on untreated cells, but that phosphorylation decreased with KU-0063794 treatment along with IL siRNA treatment.

In light of these concerns, and in line with the institutional recommendation, the *PLOS ONE* Editors retract this article.

SLL, ECH and SKK agree with the retraction. CCC, HCC, PCC, JCB and CSC did not respond.
